# Free or Forced Vaccination

**Published:** 1893-03

**Authors:** R. A. Chudleigh

**Affiliations:** Eng.


### FREE OR FORCED VACCINATION.

We admit that a vaccinated majority is competent to say to an unvaccinated minority, "We regard you as a public danger, and if you mean to dwell among us we require you to be vaccinated." We admit, too, that if the case in favor of vaccination were established beyond a doubt, the vaccinated majority would be almost justified in their act of apparent tyranny. At one time, years ago, I went very carefully over the ground of this controversy, and I found that the history of the discovery was such as to produce a strong conviction of its value. It seems that farm laborers were the discoverers of vaccination, and that Dr. Jenner having received it from them, introduced it to the world. The Gloucestershire diarymen had observed from time immemorial that their cows were liable to a pustular eruption, and that the milkers who caught this "cow pox" were proof against small-pox.

In the year 1796, Dr. Jenner was called on to inoculate a boy named Phipps with small-pox, as was then customary. He was, however, permitted by the parents to inoculate him first with cow-pox, and, after a due interval, an attempt was made to give the boy small-pox, which he successfully resisted; and a number of others subsequently underwent the same process with similar success. For a while everything tended to add repute to the operation. But as time went on, people began to accuse cow-pox of many things, of bringing disease worse than the one it cured; of so-fastening on the fifth pair of nerves that disorganized teeth, face-ache, brow-ache, and head-ache, are its regular concomitants. And, worst of all, it was accused of being no safeguard against the small-pox! The controversy became a long and a loud one; and I felt so far compelled to abandon my first position as to take up the new ground, that however successful vaccination might have been in its first introduction among the Gloucestershire dairies, it certainly failed under the test of a wider application, and fell very far short of its earlier promise.

In this frame of mind I remained until the publication of Dr. Charles Creighton's essay, and the two volumes by Professor Edgar Crookshank. Then I had to abandon my whole position, and thenceforth I regarded vaccination itself and its so-called history as one of the most singular popular delusions with which I am acquainted. Is then, vaccination to be forced or voluntary? I would reply, let those who like it have it, and believe themselves secure. And I sincerely hope that that overpowering majority (numerically) who detest vaccination will never commit the cruel despotism of preventing people from being vaccinated. At the same time, I would denounce the present custom whereby a minority is able to enforce a hated practice on its unwilling neighbors.—*R. A. Chudleigh, Eng., in Farm and Home.*

